# Implementing community pharmacy-based influenza point-of-care test-and-treat under collaborative practice agreement

**DOI:** 10.1186/s43058-022-00324-z

**Published:** 2022-07-16

**Authors:** Kenneth C. Hohmeier, Kimberly McKeirnan, Julie Akers, Michael Klepser, Stephanie A. Klepser, Christe Chen, Donald G. Klepser

**Affiliations:** 1 University of Tennessee Health Science Center College of Pharmacy, Nashville, TN USA; 2 Washington State University College of Pharmacy and Pharmaceutical Sciences, Spokane, USA; 3 Department of Pharmacotherapy, Health Outreach and External Residency Research, Washington State University College of Pharmacy and Pharmaceutical Sciences, Spokane, USA; 4 Ferris State University, College of Pharmacy, Big Rapids, USA; 5 Independent Consultant, Kalamazoo, USA; 6 College of Pharmacy, University of Nebraska Medical Center, Omaha, USA

**Keywords:** Community pharmacy, Pharmacy, Rapid diagnostic test, Point-of-care test, Influenza, Public health

## Abstract

**Background:**

Early and accessible testing for influenza with point-of-care testing (POCT) can be a critical factor for deciding to begin antiviral treatment. More than 10,000 pharmacies across the USA offer Clinical Laboratory Improvement Amendments-waived POCT for infectious diseases, such as influenza A/B. Knowledge of barriers and facilitators to large-scale POCT implementation may be useful in scaling POCT for influenza test-and-treat services (Flu POCT). The objective of this study was to explore the experiences of pharmacists who were early adopters of Flu POCT and treatment under collaborative practice agreement in community pharmacy settings.

**Methods:**

Qualitative research design with in-depth, semi-structured virtual video interviews of licensed US community pharmacists. Interview questions were derived from the Consolidated Framework for Implementation Research (CFIR). Interviewees were selected via a purposeful sampling of pharmacists who were enrolled in a nationwide clinical trial involving pharmacy-based influenza test-and-treat under a collaborative agreement. Interviews were recorded and transcribed. A deductive analytic approach was used via constructs from the CFIR.

**Results:**

Six pharmacists were interviewed. Interviews ranged from 28 to 70 min, with an average length of 46 min. Four broad themes emerged from the data, and each had corresponding subthemes and supporting quotes: influence of the Flu POCT service characteristics on pharmacy implementation, influence of factors outside of the pharmacy setting in Flu POCT implementation, factors within the pharmacy setting influencing implementation, and process of implementing Flu POCT. A novel pharmacy-based Flu POCT implementation framework is presented.

**Conclusions:**

Implementation of community pharmacy-based Flu POCT services is feasible; but, a thorough understanding of both barriers and facilitators to their implementation is needed to increase the spread and scale of these programs. Specifically, pharmacy stakeholders should focus efforts on increasing patient and provider awareness, pharmacist acceptance, leadership support, and support of health providers external to the pharmacy to improve implementation success.

**Supplementary Information:**

The online version contains supplementary material available at 10.1186/s43058-022-00324-z.

Contributions to the literature

- The community pharmacy represents an underutilized public health access point for acute infectious disease prevention and treatment as highlighted by the COVID-19 pandemic.
- Early and accessible testing with Flu POCT can be a critical factor for deciding to begin antiviral treatment.
- In many U.S. states, pharmacists may assess the patient and subsequently furnish antivirals under collaborative practice agreements using CLIA-waived POCT —expanding care access using the existing workforce.
- Existing literature suggests that Flu POCT is feasible and effective when implemented in several areas of the U.S.
- This study captures implementation perspectives from such “early adopter” pharmacies and puts forth a novel implementation framework to guide future implementation efforts.

## Background

The Centers for Disease Control and Prevention (CDC) estimate during the 2019–2020 influenza season more than 39 million people developed influenza, leading to more than 18 million healthcare provider visits. Annually, between 410,000 and 740,000 Americans are hospitalized due to influenza [1]. Additionally, complications from influenza include pneumonia and respiratory failure which can lead to worsening of chronic medical conditions. Influenza is estimated to be responsible for between 24,000 and 62,000 deaths annually [1].

Guidelines on the management of seasonal influenza from the CDC and Infectious Disease Society of America (IDSA) suggest beginning antiviral treatment within 48 h of symptom onset with suspected or documented influenza infection [2, 3]. For high-risk patients, including those who are hospitalized or may have severe consequences from influenza infection, antiviral treatment is suggested even if the preferred 48-hour window has passed [1]. Empiric treatment is also recommended for high-risk patients in situations where a community is experiencing co-circulation of influenza and SARS-CoV-2 [2].

Early and accessible testing for influenza can be a critical factor for deciding to begin antiviral treatment. Historically, viral culture laboratory testing was the standard of practice for influenza diagnosis. However, due to cost and turnaround time for results, its use is no longer recommended for initial or primary diagnosis [2]. The Clinical Laboratory Improvement Amendments of 1988 (CLIA) established quality standards for laboratory testing of specimens for diagnosis and treatment [4]. Point-of-care testing (POCT) for many infectious diseases is considered CLIA-waived, meaning they have a low risk of error and are simple to perform. POCT with rapid diagnostic tests has led to early influenza virus detection and a significant overall effect on improving patient health [5].

More than 10,000 pharmacies across the United States offer CLIA-waived POCT for infectious diseases, such as influenza A and B, and group A streptococcus [6]. In many states, pharmacists can prescribe treatment for patients after a positive test and thorough physical assessment via collaborative practice agreements or statewide protocols [7]. As defined by the CDC, a pharmacist collaborative practice agreement is a “formal agreement in which a licensed provider makes a diagnosis, supervises patient care, and refers patients to a pharmacist under a protocol that allows the pharmacist to perform specific patient care functions” [8]. Offering POCT and treatment for infectious diseases in a pharmacy setting can increase quick access to antiviral treatment, increase patient satisfaction with healthcare, improve antimicrobial stewardship, free-up physicians’ time for higher-acuity patients, and reduce unnecessary utilization of emergency departments [7]. Offering POCT in community pharmacies has demonstrated improvement in both patient health and patient-reported satisfaction [7].

According to Dulaney et al., community pharmacists reported willingness to perform POCT and recommend treatment for patients with influenza [9]. Participating pharmacists also believed they have the clinical knowledge to treat influenza and that pharmacy staff could be trained to assist with POCT services [9]. Moreover, training to perform POCT is now increasingly integrated into the Doctor of Pharmacy curricula [10].

When implementing CLIA-waived POCT in a community pharmacy setting several logistical challenges must be addressed, including establishing collaborative practice agreements, incorporating POCT into workflow, providing training for pharmacy staff, determining methods of remuneration, and following regulatory reporting requirements [11]. Barriers and facilitators to nationwide POCT implementation have yet to be explored in the published literature, but may be useful in POCT for influenza test-and-treat services. The objective of this study was to explore the experiences of pharmacists who were early adopters of Flu POCT and treatment under collaborative practice agreement in community pharmacy settings.

## Methods

We used a qualitative research design with in-depth, semi-structured virtual video interviews of licensed U.S. community pharmacists. A phenomenological approach was selected to best understand the subjective experience of community pharmacists implementing the novel service of influenza POCT (Flu POCT) and treatment with antiviral therapy prior to the COVID-19 pandemic [12]. The University of Tennessee Institutional Review Board approved this study in January 2020 (20-07309-XM).

A semi-structured interview guide was developed by experts in influenza, POCT, and community pharmacy. Questions were derived from the Consolidated Framework for Implementation Research (CFIR), and the instrument was adapted based on an expert panel comprised of clinicians (e.g., PharmD, MD), researchers, and pharmacy leadership in line with guidance from the CFIR Research Team-Center for Clinical Management Research [13]. The interview guide was first drafted by a University researcher familiar with the topic using the CFIR website (https://cfirguide.org) interview guide tool. Subsequently, the document was shared with the expert panel and the instrument was further truncated. Interviews occurred in the Spring of 2020 by two trained researchers (KH and CC). Interview sessions were audio-recorded digitally and professionally transcribed by a third-party transcription service. Interviews were conducted over virtual videoconferencing technology, recorded, and subsequently transcribed. Field notes were also collected during interviews to note non-verbal expressions and interactions and incorporated into the data analysis process.

Interviewees were selected via a purposeful sampling of pharmacists who were enrolled in a nationwide clinical trial involving pharmacy-based influenza test-and-treat under a collaborative practice agreement. Flu POCT implementation champions known to the researchers were selected as part of the stratified sample of pharmacists, which included varying geographic locations, sex, organizational roles, and practice setting (i.e., chain or independent pharmacy). Recruitment occurred over email to each of the study site organizations and continued until a point of saturation whereby no new themes emerged with subsequent focus groups [14].

A deductive analytic approach was used via constructs from the CFIR [13]. Two coders trained and experienced in qualitative research methods (KH and KM) coded two transcripts together using the CFIR codebook to ensure coding consistency, and then coded the remaining transcripts independently. A reflexive approach was used by which data analysis took place alongside data collection so that the interview guide and approach could be modified. Both coders were faculty members at US colleges of pharmacy who specialized in community practice. Once researcher was trained, credentialed, and experienced in implementation science. Transcripts and field notes were uploaded into a qualitative analytic software (NVivo, Burlington, MA), which was used to assign codes and develop themes. Lincoln and Guba's criteria for quality in qualitative research and the Consolidated criteria for reporting qualitative research (COREQ) checklist were used to ensure data collection and analytical rigor [15, 16]. A third member of the research team assisted in the resolution of disputes during the thematic analysis process. Prior to finalizing themes, participant checking occurred by sharing these pre-final themes with two of the interviewees for their feedback—however, given the limited time availability of the participants, transcripts were not returned to the participants for review.

## Results

Six participants were interviewed, one for each of the pharmacy organizations approached. Demographics for interviewees are listed in Table 1. Interviews ranged from 28 to 70 min, with an average length of 46 min, and were conducted at the participants’ homes or pharmacies. Four broad themes emerged from the data, and each had corresponding subthemes and supporting quotes (Table 2).

**Table 1:** Demographics of in-depth interview participants

Informant	Pharmacy organization classification	US region	Urban-rural classification	Experience with point-of-care testing (years)
1	Independent	Southeast	Urban	<1
2	Chain	Nationwide	Urban/Rural	6
3	Independent	Southeast	Urban/Rural	<1
4	Chain	Midwest	Urban/Rural	7
5	Independent	Southeast	Urban/Rural	2
6	Independent	Southeast	Urban/Rural	2

**Table 2:** Supporting quotes from in-depth key informant interviews

Theme	Quote	Informant #	Pharmacy organization classification	Experience with point-of-care testing (years)
Influence of the Flu POCT service characteristics on pharmacy implementation				
*Relative advantage of Flu POCT service over other pharmacy services*	“And out of the 15 (rapid influenza) tests that we did right then and there, nine, ten of them had never stepped foot in any of our stores...maybe that has that opportunity to bring in… new patients, new families into our business, and show them how we run a pharmacy.”	5	Independent	2
	“Obviously the ability to be able to attract new [patients]... [influenza testing] was the first time they had come there. So that’s definitely going to give you an advantage in the market.”	6	Independent	2
*Complexity of Flu POCT service over other pharmacy services*	“…once you get past that initially training, people become so much more comfortable.”	4	Chain	7
Influence of factors outside of the pharmacy setting in Flu POCT implementation				
*Patient needs and resources*	“The one positive thing about COVID is that it brought point of care testing more into the spotlight with pharmacies… Now, hopefully, COVID will bring patients into the pharmacy for the antibody testing. So, flu testing kind of hand in hand with that. Just marketing towards all of these different potentials that a pharmacy can offer. So, it kind of goes hand in hand and how they can complement each other.”	2	Chain	7
	“The very first day we opened the flu testing in the previous year, we had 15 tests in the first day. We hadn’t advertised it. We didn’t tell anybody. We just had a little-bitty sign.... We had a clinic next door to us, like an urgent clinic, and they were overflowing. So, people just kind of randomly walked over. They didn’t want to wait. There was 30, 40 people in their lobby, and they saw that we were doing that. And we ended up having a line on our first day, first time doing it. People just totally blown away that we were able to do this...” (informant 5, independent, 2 years)	6	Independent	2
	“…they all love it, because it’s a one stop shop, and it’s offered in a more timely fashion for them.”	3	Independent	<1 year
	“We have some rural areas up in Northern Michigan that frankly don’t have enough providers to provide care for patients. A lot of patients don’t have primary care providers, so we really set out to fill that void. I certainly feel that we’ve done that.”	4	Chain	7
	“I think just everybody being aware of the job that pharmacists and the pharmacy technicians, what we can do [would help]. A lot of people don’t realize the job and the qualifications that we have and are completely surprised by the things that we can do. And once they hear it by word of mouth, or any kind of advertising, they’re so excited about being able to come in for a short amount of time and get in, and get out with medicine if they test positive.”	5	Independent	2
	“Because we’ve had to stay open this entire time so who’s going to provide those strep tests when other places are closing and patients don’t have anywhere to go to get a rapid strep test?”	4	Chain	7
*Pharmacy networks with other healthcare settings*	“Working under a [collaborative practice agreement] with a pharmacist has not [historically] been respected as much as I wish it would be. It’s baby steps. The reason the flu thing was so readily accepted is because it’s a [collaborative practice agreement] for one drug.”	1	Independent	< 1 year
	“There’s a little bit of competition obviously… but again, a lot of those clinics are dealing with shortened hours as well. So, they’re not open until 9:00 AM and they’re not always there the whole weekend. So, it’s really a niche we still fill…. In the beginning, so several years ago, we had a few physician offices that were a little uneasy about us providing this type of care in our pharmacies, but honestly after having personal conversations with them and really [explaining] ‘it’s much like vaccinations, right?’ We’re not taking a piece of the pie; we’re just trying to expand the offering.’ So, we haven’t had any issues like that in several years.”	4	Chain	7
	“...we always do a follow-up with the individual’s primary care physician in addition to the collaborating physician. Any time that we not only prescribe a therapy to treat influenza based on a positive test, but anytime we would just test an individual, we would also reach out to the PCP just to let them know that the patient was in there and what our recommendation was.”	2	Chain	6
	“I’ve got a physician who brings his five kids here every single time instead of taking them to his office or their pediatricians and stuff like that. They roll in, all five of them, once a month, once every six weeks, whatever, trying to get a test, because one of them is sick. And they would rather do it here, because it’s faster. And financially, better for them, as well.”	5	Independent	2
	“We’ve had one office ask and say, “Hey, if you could please not do that for our patients, we’d appreciate that. We would rather see them.” We’ve had another office that refers them to us because if someone’s sick and potentially has the flu, they’re already going to have to come back to the drug store anyway so why should they even come into the office? So, some offices see that as competition, others would gladly rather have them come to us...”	6	Independent	2
*Peer pressure among pharmacy competition*	“As an independent pharmacy we have to work very hard to differentiate ourselves. One big way we do that is in [patient] care, but then also in the services we offer… It gives us an advantage over the [other pharmacies] to get folks in the door and retained.”	3	Independent	< 1 year
	“…we battle tooth and nail competing with [other pharmacies]. So, being able to provide another service [is part of the fight] we’re fighting every day. Trying to find the next thing, because reimbursements from insurance companies and from pharmacy benefit managers (PBMs) and everything right now for independent [pharmacies], it’s life or death. A lot of them are going under. We’ve seen 50% or so in the past 10 years have closed down ...So offering other services and expanding the scope of pharmacists is crucial to independent pharmacies.”	5	Independent	2
*External policies and incentives*	“I guess referring to the [collaborative practice agreement], we had to create a collaborative practice agreement with a local physician that we did with our same physician that signs off on our immunizations. That was probably the biggest delay we had was just getting all of that signed and ready to go.”	1	Independent	< 1 year
Factors within the pharmacy setting influencing implementation				
*Network and communication within the pharmacy*	“Meetings were important because all staff needs to be aware of the offering and be able to speak to it. But then also regional pharmacy leader would have calls with the pharmacist that were participating just to do a check in, to see what their barriers were, to make sure that everybody understood all the procedures that need to be in play. So, an open line of communication both between the overall leader and the pharmacist and those stores, and then also the pharmacist and the teams that they’re leading.”	2	Chain	6
	“We disseminate information through email. … we push the information out that way to our managing staff who then tell their on-site staff. We try to have morning huddles every day for focus as well as any kind of new information we might be pushing out to get to our pharmacy technicians and some of the other staff at each store. So, we have a little bit of a flow that starts from upper management and kind of trickles down. Phone calls as well, but the easiest way to get things processed is through email.”	3	Independent	< 1 year
	“So, at [our pharmacy chain] we have a clinical division, and then we have almost like a business division. I don’t even want to call them silos, because we work so closely together, but between business and clinical. In terms of implementation, I don’t think there was ever a question from the business side that this was the right thing to offer. We communicate it to both sides because we think it’s important for the entire leadership team to know what we offer”	*4*	*Chain*	*7*
*Culture of the pharmacy*	“[We’re] highly efficient. We run one pharmacist and four technicians managing 500+ patients in a very different model; My whole model is just a different deal.”	1	Independent	< 1 year
	“We’re all looking for ways to matter to our patients and to make sure we’re going above and beyond for them. …I think that [our pharmacy] has a great culture for implementation. …we have implemented transitions of care, we’ve implemented a new adherence packaging program, so our staff is always prepared and ready for new things. [The pharmacy staff] are very flexible, and I think there’s just a great culture for implementation within all the [pharmacies]. …I think also, the job satisfaction that goes into it for our pharmacists as well, has been a huge reward. So, this always sounds funny and a little weird saying, but we get excited when we get those positive tests. Not because someone’s ill, but because we can help someone. We found someone that needs therapy that, you know what? In a few days, they’re probably going to start to feel a lot better and maybe they wouldn’t have gotten that therapy had we not been available for the service. So incredibly rewarding.”	3	Independent	< 1 year
*Implementation climate of the pharmacy*	“We’re very adaptive I guess at [our organization]. It doesn’t take months and months and months to make a change, if we need a change, we can get that approved and completed quickly.”	4	Chain	7
	“[Flu POCT is] high priority... We are really working hard on how we expand, and both provide more services to our patients as well as looking to get reimbursed by insurances for that. So, we’re evaluating medical billing platforms currently to allow us to practice at the top of our license. There’s a whole lot of things that we’ve got going on, and I don’t need my pharmacists just spending all day staring at a computer and checking… You’re going to be able to come to [our pharmacy] for your prescriptions, testing supplies, and if you’re not feeling well, come over here and let us test you for Flu or Strep or whatever else you want to do. Save you some time and an office co-pay and get you out of there. I think it’s just another piece of the puzzle. I think with having those other services in place to free up pharmacists’ time, we have been doing Med Sync and tech product verification for so long that’s kind of just become part of our practice, and the pharmacists are having more and more time. So, don’t think it’s going to be a burden or an inconvenience to step away and spend a little bit of extra time with a patient.”	3	Independent	< 1 year
	“So, I think if anything, the times we’re in right now, have made us see that this was the right thing to decide to do five or seven years ago. It’s going to make our clinical programs stronger because we’ve got to help our communities through this. …How do we fit it into this new realm of healthcare? I think there’s so much of that, the unknown right now, and while it’s a scary time, I think that pharmacy is going to actually come out stronger clinically because we have to.”	4	Chain	7
	“…in pharmacy, we had just experienced initiating something new probably five years before that with vaccines, so it was just revisiting ‘ok, how are we going to adapt for change?’ It’s really change leadership [strategies], that we used more or less.”	4	Chain	7
	“Anytime you add additional workload into a pharmacist day there’s always the initial stress of, ‘How do I do this,’ and ‘This is one more thing I need to do.’ But I think the satisfaction, once you can pull away from dispensing and really get that one-on-one time with a patient that the point of care testing offers, it’s very rewarding. I think it’s a good balance for pharmacists.”	2	Chain	6
*Pharmacy readiness for implementation*	“When we were pitching the program [our top leadership] were full of recommendations. ‘Let’s get you in touch with marketing so we can make flyers about this service and get store posters out there letting the patients know.’ Of course, because you have to drive people in to get the tests. So, they were definitely supportive in that aspect, as pharmacy isn’t always used to creating and doing marketing pieces like that. So, we had to get in touch with other departments in the company.”	4	Chain	7
	“…by the time the analyzers were actually received in stores, we were only just a matter of two, three weeks away from everything happening with COVID...that was one of the barriers, really just the timing of all the pieces coming together in an impactful way for this flu season.”	6	Independent	2
	“So, word of mouth honestly goes a long way to be quite honest. Social media’s been huge. We had a big social media push in January. Someone got a test and they put it on social media and it just exploded. Media was everywhere. So, it’s funny what social media does to things. But when it came around this year, we started to press a little more... our goal was to kind of get going, get it spread out... we started with some advertising...We do radio commercials, we do billboards in town. All the old school stuff, it still works great where we’re at.”	4	Chain	7
	“I thought it was a good refresher… I did feel compelled to do that, and there was a section in there on collaborative practice. The final section of it was collaborative practice agreements, new billing mechanisms, and doing clinical stuff and getting paid for it. That was helpful. So, I’d recommend that for anybody. That was good."	1	Independent	< 1 year
	"[The pharmacists] got certified through a Point of Care Testing certification program. All the pharmacists in the company. And [a local University] facilitated and organized that.	3	Independent	< 1year
	“[Pharmacy leadership] created an in-house training… We went through [a national point-of-care testing certificate course] Train the Trainer Training. That’s a lot of training there. They all had to go through [in-house training and national certification]. Every flu season is new because you haven’t tested people for potentially six months depending on flu activity. So, we like to do refreshers. Certain modules that they have to complete or videos they have to watch.”	4	Chain	7
Process of implementing Flu POCT				
*Planning*	“Oh my goodness, we were pretty detailed with it. Lots of manuals, lots of policies and procedures, and entire things spelled out because it was so new to everyone. So, we had everything was spelled out.”	4	Chain	7
*Engaging*	“I think I’d be here an hour telling you how much I love the fact that my technicians get to do this. It’s, again, phenomenal for me to watch them grow in their position, as well... With these technicians, it’s my job to train them to do exactly how I would do it, how I would approach things, how I would treat the patient... if you think about it, with an everyday patient picking up a prescription... the point of contact is that technician. So, they’re probably just as comfortable with that technician that’s always seeing them than they are anybody. So, to have that person... be able to sit back with them, take their temperature, go through questions and go through everything, the basic details on the data sheet, that’s more comfortable to them, and seeing someone they see every day. Or every time they come by the pharmacy.”	3	Independent	< 1 year
	“I think that’s our biggest hurdle. Having everyone on board. In the pharmacies that have a pharmacist and technicians that are all about it, and pushing it, and helping, and providing it, and talking about it, and enjoying it. If you’re doing the testing and you’re bitter and you’re grumpy, people aren’t going to come back anyway, just because they don’t want to be around you. So having everyone on board, I think is the biggest hurdle for our pharmacy.”	5	Independent	2
*Executing*	“...even with what we experienced with COVID-19 kind of putting a stop to it, we were still more than almost triple our numbers from the year before. And we thought we did great the year before. We were impressed with our first-year numbers. But to see, even being cut probably a couple months, we were over triple the amount of tests.”	2	Chain	6
*Reflecting and evaluating*	“[Flu POCT implementation] weighs very high. It’s one of our goals for the year. So, doing more implementation of point of care testing will definitely be a high evaluator of my performance.”	2	Chain	6
	“We probably should set better goals or set an additional number that we want to treat. Right now, it’s all passive in nature that they come to us after we promote it, but we really haven’t set any goals.	6	Independent	2
	“We don’t necessarily have goals as far as a metric to hit. We put it out there that all stores will be trained coming into this flu season. But as far as specific goals, it really is going to depend on where you are and what the flu season looks like. So it’s hard to put a specific number when we don’t know what flu season may look like.”	2	Chain	6
	“I think at least covering the cost, the expenses. Seeing that we are doing tests in every store to make it beneficial, not just as a monetary standpoint but also make sure that we’re serving our purpose of helping the community in identifying those cases. So I think a combination of, it’s not a money pit, and also that we truly are finding patients that can benefit from this program.”	4	Chain	7
	“I think the return of investment is really important. We’re paying for our pharmacists to be trained. So if we can get enough tests, at least to break even on that training… I think even if it’s just increasing awareness that your pharmacy is somewhere where you go to get this type of service done, that adds a lot as well. I think both of those could be used to measure the overall success of this. And then also helping the patients of the community. If we can detect flu earlier than waiting for them to go see their PCP, overall, just trying to limit exposure rate in our communities is huge.”	2	Chain	6
	“[Success is] being able to practice at the top of their license, being able to provide a high level of patient care and being able to bring in additional revenue streams.”	6	Independent	2
	“Our goals were really to improve quality of health and expand services that were provided to the patients in certain communities, especially communities that maybe had lower amounts of PCP. We have some rural areas up in Northern Michigan that frankly don’t have enough providers to provide care for patients. A lot of patients don’t have primary care providers, so we really set out to fill that void. I certainly feel that we’ve done that.”	4	Chain	7
	“We try to dig and dig and dig, whether it be in person, after the test, before the test, social media. Anything we possibly could to get as much data on how successful, or how appropriate, or how convenient, efficient that this process would be for our patients and for our communities…Was it worth it? Would people actually use it? Would it be helpful to them? …our goal was to kind of get going, get it spread out. It kind of got to where other people around the state, around the city were starting to think about doing it.” (informant 5, independent, 2 years)	5	Independent	2
	“Patient dissatisfaction I think would be an unsuccessful marker. I guess my goal is not necessarily right now a certain number of tests, but it would just be good data collection and good reporting so I can see, of whatever test we end up doing, what it’s meant to the company and what it’s meant to the patients.”	3	Independent	0

### Theme 1: Influence of the Flu POCT service characteristics on pharmacy implementation

#### Relative advantage of Flu POCT service over other pharmacy services

This subtheme centered on participants’ perceptions of the advantage of implementing Flu POCT in relation to other pharmacy services. Participants opted to implement pharmacist-delivered Flu POCT over spending time in other pharmacy services for reasons of professional satisfaction by means of providing direct patient care, ability to serve new patients within the pharmacy, and offering services not offered at other pharmacies. Participants noted unanticipated facilitator for implementation was that the service primarily attracted new patients to the pharmacy.

#### Complexity of Flu POCT service over other pharmacy services

Flu POCT complexity centered on workflow integration, sample collection, and test supply procurement. Workflow integration of Flu POCT test-and-treat was customized to each pharmacy and required small changes, mostly in preparing the team how to handle the time when the pharmacist stepped away from their computer terminal and away from drug dispensing workflow. It was noted that these changes were similar to those made when first integrating vaccinations into workflow. Participants indicated initial staff pharmacist hesitance to perform sample collection via intranasal swab, but this was overcome with training and increasing experience. A major hurdle in the implementation of Flu POCT was obtaining the testing device, something not normal to pharmacy inventory, and this delayed implementation for some participants. In these instances, the gap between procurement of the Flu POCT device and pharmacist training served as an additional barrier as there became a need to refresh pharmacists on previously taught materials while awaiting Flu POCT machines and supplies.

### Theme 2: Influence of factors outside of the pharmacy setting in Flu POCT implementation

#### Patient needs and resources

Both patient needs and barriers to meeting those needs were discussed. Specific to patient needs, participants noted that patients prioritize convenience, speed of care, and service access, but were mostly unaware of the pharmacy’s ability to meet these needs with the Flu POCT service. Participants articulated most patients using the Flu POCT service were not patients of the pharmacy, but had come to the pharmacy based on word-of-mouth recommendations or advertising. A resounding barrier among participants was the lack of patient awareness that pharmacists can perform Flu POCT. Similarly, for participants who had performed Flu POCT for one or more previous seasons, they noted how word of mouth, advertising, referrals, and social media facilitated awareness of how this service met patient demands for convenience and speed of care. It was also noted how the SARS-CoV-2 pandemic raised awareness of the expanded role of the pharmacist and how this may facilitate further expansion of the Flu POCT.

#### Pharmacy networks with other healthcare settings

This subtheme centered on the degree to which the pharmacy’s network of prescribers, hospitals, and medical offices impacted implementation. Participants noted in some cases there was initial resistance to participants performing Flu POCT, but that this was usually limited to a single medical practice or prescriber and overcome with proactive communication and time. Related to communication, participants emphasized the importance of communicating Flu POCT care plans to the patient’s primary care physician over telephone or fax as a key policy of their service.

#### Peer pressure among pharmacy competition

Broadly participants discussed the challenges of community pharmacy’s current business model and the need to expand or develop new patient care services offered. Participants at independent pharmacies noted the need to offer services not seen by the national pharmacy chains to differentiate themselves. Participants at chain pharmacies noted that they too were in competition, but with both the other chain pharmacies and independent pharmacies.

#### External policies and incentives

Both future third-party reimbursement potential and advancing scope of practice were facilitators for Flu POCT. Participants noted that continued national and state-specific conversations around third party payment models drove interest from pharmacy leadership as a supplement to cash-based payment models. Also, the provision of a prescription antiviral after receiving a positive flu result and corresponding patient assessment (rather than the result alone without treatment) was essential to program success; therefore, working within states allowing expanded scope of practice or broad collaborative practice agreement rules facilitated both implementation and scaling of services. Participants noted that even with the regulatory ability to both test and treat influenza, there sometimes existed a barrier in finding a collaborating physician to oversee the program and partner under a collaborative practice agreement.

### Theme 3: Factors within the pharmacy setting influencing implementation

#### Network and communication within the pharmacy

This subtheme refers to the informal and formal communication strategies used by participants within their pharmacy. Generally, independent pharmacies used a more informal communication method (e.g., text messages, unscheduled conversations) to implement and sustain Flu POCT, whereas chains used existing formal communication methods (e.g., messaging campaigns, scheduled and routinized emails, scheduled visits). Leadership and front-line communication were noted to be critical to success. Communication across settings generally occurred over email and occasionally telephone. Face-to-face communication happened most often between front line staff members or between members of the leadership team and was due to the close physical proximity of those individuals. However, these siloed communication loops were not found to serve as a barrier for implementation.

#### Culture of the pharmacy

The culture of pharmacies implementing Flu POCT was described as innovative, highly efficient, and clinically focused. Participants considered their organizations as “leaders of the pack,” and expressed pride in their innovative approaches to the practice of pharmacy. Overall the pharmacies were noted to have characteristics in line with Rogers’ definition of Early Adopter and Innovator organizations [17]. Organizational priorities aligned with advanced and clinically focused services, and front-line staff reflected those priorities. Participants were noted to proactively seek out new opportunities to provide patient care as it positively affected their quality of work life (QOWL). These pharmacies also were concurrently implementing other novel patient care services concurrently with Flu POCT. Participants noted the synergetic interplay between the other clinical services offered at the pharmacy, noting that more and varying clinical services offered at the pharmacy facilitated implementation, rather than served as a distraction or competition for Flu POCT (e.g., Flu POCT services complimented vaccines and medication therapy management services).

#### Implementation climate of the pharmacy

The implementation climate of the pharmacy is the “shared perception among intended users of an innovation, of the extent to which an organization's implementation policies and practices encourage, cultivate, and reward innovation use” [18]. Like pharmacy cultures, organizational climates were noted to be supportive of innovation with support for new initiatives. Participants who had organizational leadership roles noted the importance of implementing new patient care services as a part of their overall organizational strategy. Participants felt their pharmacy organizations prioritized initiatives which embraced new roles in public health. Policies and procedures from past, successful patient care service implementations enabled Flu POCT implementation.

Incentives were sometimes provided by organizations to support implementation—but this was not seen across all pharmacies. Reasoning to not specifically incentivize their staff centered on relying on the intrinsic motivation of pharmacists to take on new clinical services for their own quality of work life and professional satisfaction, and this was found to be a sufficient incentive for those pharmacies using that incentive. This concept of professional satisfaction was articulated by both management and front-line pharmacists.

Participants noted that Flu POCT was overall fully compatible with existing workflow, work tasks, and physical layout of the pharmacy; although some minor workflow adaptations were required. Some specific physical aspects required included a private patient care room located in proximity or attached to the pharmacy department itself. It was also compatible with pharmacist views of their own professional responsibilities and professional capabilities.

#### Pharmacy readiness for implementation

Participants noted readiness for implementation revolved around a few core items: advertising and patient awareness, training and education, and testing equipment. Pharmacies with longer experience providing Flu POCT had achieved each of these items; similarly, those who had not yet begun testing (despite a high degree of willingness) lacked the majority of the three.

Engagement from top-level management teams was a key facilitator for implementation. This was especially true given the unique circumstance whereby patients were unaware of the pharmacist-delivered service. Marketing and advertising were two areas in particular where collaboration between departments was required and was largely organized by upper management and leadership. This included social media, flyers, bag stuffers, billboards, radio advertisements, and store signage. Furthermore, top-level management and leadership also organized and facilitated training & education for their pharmacy staff and procured testing supplies and equipment.

Access to both the testing equipment, supplies, and supporting training and education were also noted to be crucial elements for successful implementation. As most pharmacy suppliers do not carry POCT equipment or supplies, new supply chains or ordering procedures needed to be developed for most pharmacies—causing several pharmacies to have initial delays in implementation. Similarly, despite having foundational knowledge in therapeutics and pathophysiology (including infectious diseases, medical microbiology, and public health), most community pharmacy practices do not involve routine use of the knowledge required for Flu POCT. Therefore, participants referred to the value of “refresher” courses in infectious diseases, laboratory diagnostics, and related administrative topics (e.g., credentialing, regulatory policies, medical billing).

### Theme 4: Process of implementing Flu POCT

#### Planning

Planning was noted to be detailed and involved the creation of new policies and procedures in line with other pharmacy-based services. Planning was mostly performed in a top-down manner, with pharmacy ownership or corporate leadership being responsible for developing and enforcing these policies and procedures.

#### Engaging

Generally, engaging participants and their support staff in Flu POCT implementation was straightforward and not resource-intensive. Given recent shifts within the pharmacy profession toward services that offer direct patient care, pharmacy staff enthusiastically participated in the implementation and scaling of the program. Though, several interviews noted a persistent minority of those who do not embrace change to their work tasks like Flu POCT, despite social norms supporting Flu POCT,

#### Executing

Participants who had already implemented POCT in years prior noted that despite the impact of COVID-19 on the flu season, patient demand grew. For those just beginning the implementation, they noted how positive patient feedback facilitated service rollout and staff enthusiasm in implementation.

#### Reflecting and evaluating

Goals and measures of success for Flu POCT varied depending on the maturity of the pharmacy’s Flu POCT service. Some pharmacies set a goal of a specific number of tests, while others found setting such a goal difficult because of the variability of the flu season year-to-year. When discussing financial goals, participants articulated their goals of expanding current patient care service offerings and “breaking even” on the cost of the program.

Generally, most measures of success centered around improving patient care and diversifying pharmacy service offerings.

Participants noted another goal was to set up a lean experiment model centered on the voice of the consumer (i.e., to test the service using the minimum amount of resources to see if there was enough patient demand in the community). Given the novelty of Flu POCT as a service and of a community pharmacy as an acute care access point, participants used the service to test patient demand and feasibility.

## Conceptual framework of implementation facilitators to community pharmacist-delivered Flu POCT test and treat program implementation

**Fig. 1:**
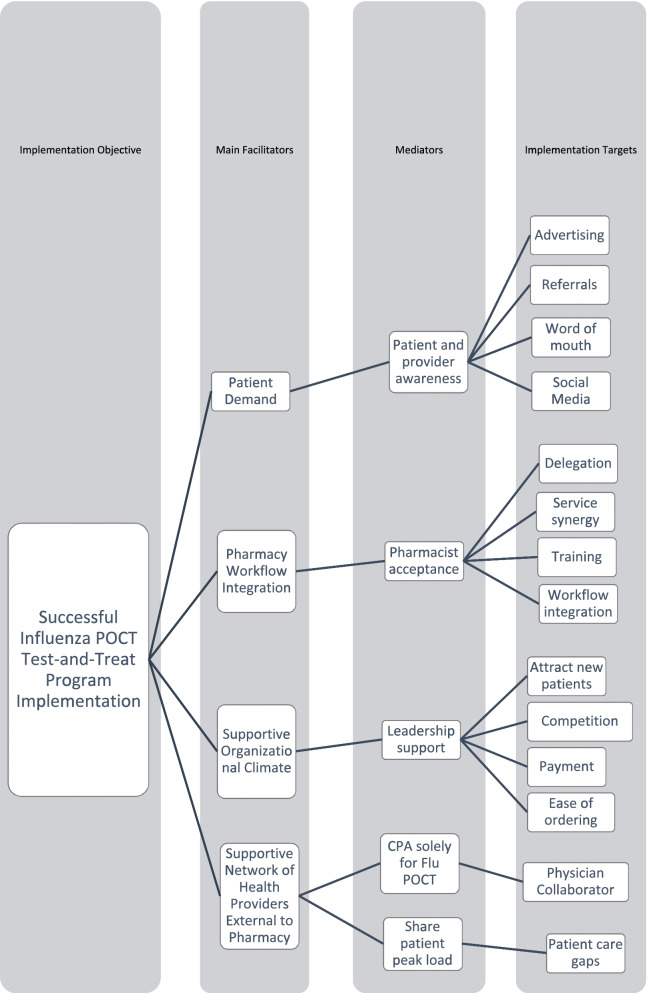
Conceptual framework of implementation facilitators to community pharmacist-delivered influenza point-of-care test and treat program implementation. POCT, point-of-care testing

Figure 1 represents the resulting conceptual framework developed from the semi-structured interview results. Four main facilitators were present: *patient demand*, *pharmacy workflow integration*, *supportive organizational climate*, and *supportive network of health providers external to the pharmacy.* Each of these main facilitators was mediated by at least one other variable, which included (a) *patient and provider awareness*, (b) *pharmacist acceptance*, (c) *leadership support*, (d) *protocol-driven collaborative practice agreemen*t *solely for Flu POCT test-and-treat*, (e) and *sharing patient load during peak season/outside normal clinic hours.* Of note, these latter two mediators (mediators “d” and “e”) related to the broader concept of built trust between the pharmacist and other healthcare providers. The first of these two mediators, *protocol-driven collaborative practice agreement solely for Flu POCT test-and-treat*, refers to the overseeing physician’s comfortability with delegating responsibilities to a pharmacist.

The simplicity of the Flu POCT protocols used by the participants, including patient assessment, laboratory result interpretation, and prescribing guidelines, facilitated trust where prior to this collaborative practice agreement there was none. Although it is standard practice for nurse practitioners and physician assistants to work under these collaborative agreements, the inclusion of a pharmacist in such an agreement is novel and therefore may represent additional risk or liability at first to a physician who previously had not collaborated with a pharmacist. The second mediator, *sharing patient load during peak season/outside normal clinic hours*, was a downstream mediator from the use of the collaborative practice agreement. Once the Flu POCT service had begun and patient experiences became known in the community, other clinicians within the community increased their trust in the service such that they saw an opportunity to refer low complexity cases to the pharmacist in a way that closely mirrors how clinicians refer routine vaccinations to community pharmacies presently to offload low complexity workload. Finally, a series of implementation targets are listed within the framework.

## Discussion

Emerging evidence suggests that community pharmacies may serve as a key, underutilized care access point for acute infectious disease prevention, testing, and treatment [7, 19–21]. However, widespread implementation and scale of such community pharmacy-based POCT services, similar to what has been seen in pharmacy-based immunization services, will be dependent on a thorough understanding of barriers and facilitators to the service. To this end, we conducted interviews with pharmacists in a nationwide sample as part of a contextual inquiry to elucidate these barriers and facilitators. In our study, pharmacy sites ranged from local independent pharmacies to nationwide chains with differing patient populations. The results provide insights into the pharmacist’s point of view on Flu POCT in a community-based setting just prior to the COVID-19 pandemic. Such data will be of use to payers, researchers, and healthcare professionals as they seek to identify new ways to bolster public health efforts during and beyond the ongoing pandemic.

The study also provides deeper insights to existing published research and expert opinion which can be found in the literature. Pharmacist’s growing acceptance of POCT services can be represented by the over 5,000 pharmacists who have been credentialed by a nationally recognized POCT certificate program through 2020 [19]. POCT service workflow compatibility has also been researched previously and found to be in line with time spent on other clinical pharmacy activities, including pharmacy-based immunization services (ranging between 2.6 and 12.7 min) [19, 20, 22]. According to participants in this study and in the previously published literature, obtaining a collaborative practice agreement continues to be a substantial barrier due to a “unfamiliarity with statues and pharmacist capabilities; although, in general, physician-pharmacist collaborative practice under a collaborative practice agreement continues to grow across the USA [8, 23]. To this end, the study also presents a novel conceptual framework which ties salient pieces of Flu POCT implementation together. Of note, researchers and those responsible for implementing these services within community pharmacies can use the “Implementation Targets” within the framework as either a checklist for assessment or outline for planning.

The potential public health benefit of the community pharmacy is primarily that of patient accessibility [24]. Accessibility in this context refers to geographical location, hours of operation, and overall cost of the service. Based on a previous study, 92% of the US population live within 5 miles of a pharmacy [25]. Moreover, patients frequent their community pharmacist more often than their primary care physician [26]. A 2020 study also reported that 43.9% of patients sought care from a pharmacy outside of doctor’s office hours [21].

Community pharmacy-based POCT’s impact on public health has been studied to a great extent in the published literature. Several efficacy studies have shown the benefits of POCT test-and-treat in the community pharmacy setting. Results from a collaborative physician-pharmacist Flu POCT test-and-treat for patients presenting with influenza-like illness (ILI) across 55 pharmacies demonstrated the service’s feasibility and acceptability during the 2013–2014 influenza season. In total, 121 patients were screened within the program, 35% of whom did not have a primary care physician and about 40% were seeking care outside of normal clinic office hours. Of note, 37% of patients did not meet eligibility for the service according to the collaborative practice protocol and were referred to their physician or an urgent care for further evaluation. Just over 1-in-10 patients were provided oseltamivir based on physical assessment and positive Flu POCT result, and at follow-up, only 3% of those patients reported worsening symptoms and were referred for further medical care [27]. Although racial and ethnic data was not available in this study, these findings do indicate that a substantial proportion (about 2-in-5) had access to this service despite an existing barrier (e.g., lacking a primary care physician or being unable to seek care during normal business hours). In general, retail care settings have generally increased care access, at lower costs, with similar quality across select preventive and acute care conditions and have been [28, 29]. When looking specifically at retail clinics, the largest age group using these services are between 18 and 44 years old who lacked a primary care provider and that these settings are primarily located in non-medically underserved areas [30]. In comparison, community pharmacies are found almost equally across both medically underserved and non-underserved areas—possibly representing greater care access to a more diverse patient population [31, 32]. However, given the nascency of these pharmacist-provided collaborative care services (i.e., Flu POCT), the specific impact of health equity and disparities has yet to be investigated.

Similarly, another study in 2016 community pharmacy-based study resulted in 40% of patients testing positively for influenza using POCT in combination with a thorough physical assessment. Of those patients, 63% were prescribed oseltamivir under a collaborative practice agreement directly from the pharmacy and over-the-counter medications were provided to 85% of patients for symptom management [33]. Pharmacists were able to follow up with 56% of these patients, all of whom were recovering with a noted decrease in flu symptoms. In a more recent study performed across 6 states and 6 pharmacy chains, 93.8% of the flu-positive patients were able to obtain an antiviral prescription per collaborative practice agreement and 88% of negative tests received over-the-counter recommendations [19]. Of these patients, 80% had improved symptoms within 24–48 h of receiving a Flu POCT.

There were limitations to this study. Given its qualitative research methodology approach, generalization of the results to the entire population of community pharmacists is not possible. A future, follow-up study which uses the present study’s results to develop and disseminate a cross-sectional questionnaire would be best suited in establishing generalizability. Qualitative research is also highly dependent upon the data analysis team and their qualifications. In the case of the present study, this limitation was mitigated in part by both data coders (KH and KM) having been trained in and previously published on qualitative research methodology, while also being licensed community pharmacists with POCT experience.

## Conclusion

Implementation of community pharmacy-based Flu POCT services, which include prescribing of antivirals under collaborative practice laws, is feasible; but a thorough understanding of both barriers and facilitators to their implementation are needed to increase the spread and scale of these programs. Specifically, pharmacies should focus efforts on increasing patient and provider awareness, pharmacist acceptance, leadership support, and support of health providers external to the pharmacy to improve implementation success.

## Supplementary Information

**Additional file 1.** Semi-Structed Interview Guide.

## Data Availability

Given the qualitative data presented here, size and characteristics of the sample and disclosure of data and material will compromise the confidentiality of the research participants. To protect the anonymity of their responses, data and material will not be made publicly available. However, requests for data may be made to the corresponding author.

## Abbreviations

PBM: Pharmacy benefits manager
CLIA: Clinical Laboratory Improvement Amendments
POCT: Point-of-care test
CFIR: Consolidated Framework for Implementation Research
CDC: Centers for Disease Control and Prevention
IDSA: Infectious Disease Society of America
COREQ: Consolidated criteria for reporting qualitative research
QOWL: Quality of work life
ILI: Influenza-like illness
