# Supplementary material for: Implementing community pharmacy-based influenza point-of-care test-and-treat under collaborative practice agreement
Source: Implement Sci Commun. 2022 Jul 16;3:77. doi: 10.1186/s43058-022-00324-z (PMC9287716; doi:10.1186/s43058-022-00324-z)
Supplement: Supplementary file 1 — Additional file 1. Semi-Structed Interview Guide. [file 43058_2022_324_MOESM1_ESM.pdf]

Influenza Point-of-Care Testing (POCT) Implementation in Community Pharmacies

Interview Guide

Demographics

1. What is your profession/role? How long have you been in that profession/role?
2. What is your title at your organization?
3. How long have you been with your organization?
4. Does your organization serve an urban, suburban, and/or rural patient population?
5. Would you classify your pharmacy as high-, medium-, or low volume in terms of the number of prescriptions filled per day?
    a. Why do you say that?
6. Approximately how many locations does your organization have?
7. How would you describe the population your organization serves – i.e. urban, suburban, small town, rural, college town, etc.? (May need to clarify so that it's specific to the respondent's location, especially if that location is part of a larger chain)

Outer Setting

Patient Needs & Resources

1. Have you elicited information from patients regarding their experiences with pharmacist-administered influenza point-of-care testing (hereafter referred to as flu POCT)?
    - What are their perceptions of pharmacits providing flu POCT?
2. Have you heard stories about the experiences of participants with the intervention?
    - Can you describe a specific story?

Peer Pressure

1. To what extent would implementing the flu POCT provide an advantage for your pharmacy compared to other pharmacies in your area?
    - Is there competition with pharmacy-based convenient care clinics, primary care (physician, NP, PA, etc.) offices, urgent care, etc. and the service your providing? Why or why not?
    - How much do pharmacists see these others as willing to work collaboratively vs. seeing pharmacists as a threat and thus not wanting to work with pharmacists?
    - Is there something about flu POCT that would bring more individuals into your pharmacy, instead of another one in your area?

Inner Setting

Structural Characteristics

1. How did the infrastructure of your pharmacy (social architecture, age, maturity, size, or physical layout) affect the implementation of flu POCT?
    - How did the infrastructure facilitate/hinder implementation of flu POCT?
    - How did you work around structural challenges?
2. What kinds of infrastructure changes were needed to accommodate flu POCT?
    - Changes in scope of practice? Changes in credentialing / privileging? Changes in formal policies? Changes in information systems or electronic records systems? Other?
    - What kind of approvals were needed? Who needed to be involved?
    - Can you describe the process that was be needed to make these changes?

Networks & Communications

1. Can you describe your working relationships with your provider colleagues in your local area (physicians, nurses, etc.)?
2. Please describe interactions between your pharmacy and external health-systems or integrated provider networks when collaborating on care for an individual patient (e.g. record sharing, callbacks, therapy change requests)?
    - Can you expand on power dynamics, respect and trust levels with providers in these large organizations as it relates to flu POCT
3. How do you typically find out about new information, such as new initiatives, accomplishments, issues, new staff, staff departures?
4. Are meetings, such as staff meetings, held regularly?
    - How important were these for implementing the flu POCT there?

Culture

1. How do you think your organization's culture (general beliefs, values, assumptions that people embrace) will affect the implementation of flu POCT?
    - Can you describe an example that highlights this?

Implementation Climate

Relative Priority

1. What kinds of high-priority initiatives or activities are already happening in your setting?
    - What is the priority of getting flu POCT implemented relative to other initiatives that are happening now?
    - Will the implementation of flu POCT conflict with these priorities?
    - Will the implementation of flu POCT help achieve (or relieve pressure related to) these priorities?

Organizational Incentives & Rewards

1. What kinds of incentives are there to help ensure that the implementation of flu POCT is successful?
    - What is your motivation for wanting to help ensure the implementation is successful?
2. To what extent do you think your supervisor will consider your role in this implementation in your (next) evaluation? In his/her regard for your work or role?
    - Will your role be considered differently if flu POCT is perceived to be successful vs. if it is not perceived to be successful / needs additional tweaking?

Leadership Engagement

1. What level of involvement has leadership at your organization had so far with implementation of flu POCT?
   - o Who are these leaders? How do attitudes of different leaders vary?
   - o What kind of support have they given you? Can you provide specific examples?

Available Resources

1. Do you expect to have sufficient resources to continue implementing and administering flu POCT?
   - o [If Yes] What resources are you counting on? Are there any other resources that you received, or would have liked to receive?
   - o What resources will be easy to procure?
   - o [If no] What resources are needed, but will not be available?

Characteristics of Individuals

Knowledge & Beliefs about the Intervention

1. How do you feel about pharmacist-delivered flu POCT at your setting?
   - o How do you feel about the plan to implement the intervention in your setting?
   - o Do/did you have any feelings of anticipation? Stress? Enthusiasm? Why?

Process

Planning

1. Can you describe what is/was the plan to implement flu POCT at your pharmacy?
   - o How detailed is/was the plan? Who knows about it? Is the plan overly complex? Understandable? Realistic and feasible?
   - o What is your role in the planning process?
   - o Who is involved in the planning process? What are their roles?
   - o Are the appropriate people involved in the planning process? How engaged are they?
   - o How do you plan to track the progress of implementation based on your plan?
   - o What if you have to modify or revise your plan due to barriers, errors, or mistakes?
   - o How will your organization perceive modifications and revisions? *(Probe somehow without biasing the respondent: will the organization see changes in a positive, growth-oriented mindset (e.g. learnings, opportunities for improvement, iteration) or in a negative mindset (i.e. failures, mistakes, need to assign blame)).*

Engaging

Key Stakeholders

1. What steps have been taken to encourage individuals to commit to performing flu POCT?
   - o Which patients will you target?
   - o How will you approach them?
   - o What information will you give them?
   - o How frequently and how will you communicate with them?

Executing

1. Has flu POCT been implemented according to the implementation plan?
    - [If Yes] Can you describe this?
    - [If No] Why not? What remains to be done? What barriers / hurdles are keeping these things from being done?

Reflecting & Evaluating

1. To what extent has your organization/unit set goals for implementing flu POCT?
    - How will goals be communicated in the organization? To whom will they be communicated?
    - What are the goals? How and to whom will they be communicated?
2. What would your organization need to see in order for flu POCT to be considered successful?
